# (4*Z*)-1-Methyl-4-[(2*E*)-2-(4-methyl­benzyl­idene)hydrazin-1-yl­idene]-3,4-dihydro-1*H*-2λ^6^,1-benzothia­zine-2,2-dione

**DOI:** 10.1107/S1600536812039529

**Published:** 2012-09-22

**Authors:** Muhammad Shafiq, William T. A. Harrison, Islam Ullah Khan, Ejaz

**Affiliations:** a Department of Chemistry, Government College University, Faisalabad 38000, Pakistan; b Department of Chemistry, University of Aberdeen, Meston Walk, Aberdeen AB24 3UE, Scotland; c Materials Chemistry Laboratory, Department of Chemistry, Government College University, Lahore 54000, Pakistan

## Abstract

In the title compound, C_17_H_17_N_3_O_2_S, the dihedral angle between the aromatic rings is 6.3 (5)° and the C=N—N=C group is statistically planar [torsion angle = 179.8 (8)°]. The conformation of the thia­zine ring is an envelope, with the S atom displaced by 0.823 (9) Å from the mean plane of the other five atoms (r.m.s. deviation = 0.012 Å). In the crystal, C—H⋯O inter­actions link the mol­ecules into *C*(5) chains propagating along [101]. The chains are consolidated by weak aromatic π–π stacking between the benzene and toluene rings [centroid-to-centroid separation = 3.826 (5) Å and inter­planar angle = 6.3 (4)°].

## Related literature

For the synthesis and biological activity of the title compound and related materials, see: Shafiq, Zia-ur-Rehman *et al.* (2011 ▶). For related structures, see: Shafiq, Khan *et al.* (2011 ▶); Shafiq *et al.* (2012 ▶). For C—H⋯O inter­actions, see: Steiner (2006 ▶). For graph-set nomenclature, see: Bernstein *et al.* (1995 ▶).
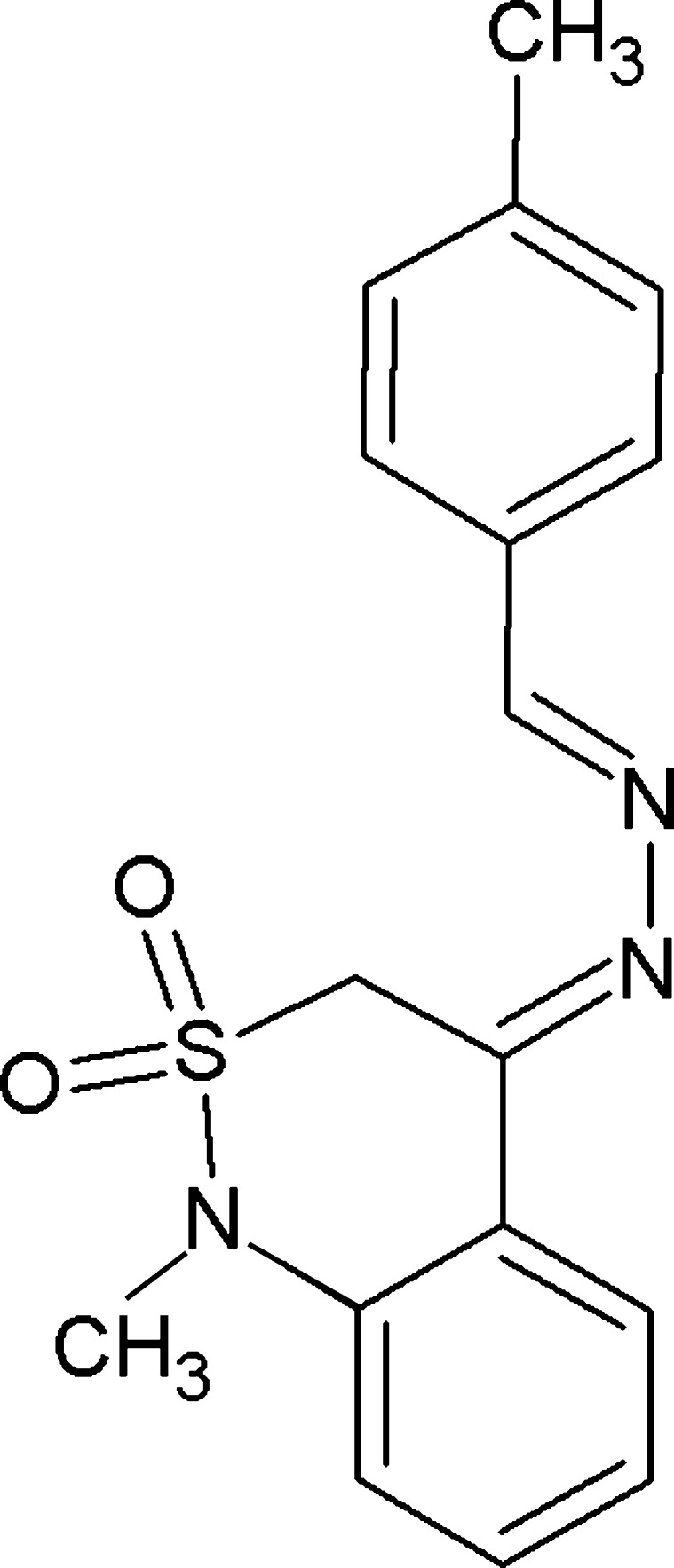


## Experimental

### 

#### Crystal data

C_17_H_17_N_3_O_2_S
*M* *_r_* = 327.40
Monoclinic, 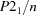

*a* = 7.899 (1) Å
*b* = 25.061 (3) Å
*c* = 8.1743 (11) Å
β = 101.114 (9)°
*V* = 1587.8 (3) Å^3^
*Z* = 4
Mo *K*α radiation
μ = 0.22 mm^−1^
*T* = 296 K
0.45 × 0.21 × 0.09 mm

#### Data collection

Bruker APEXII CCD diffractometer
13483 measured reflections
2877 independent reflections
1897 reflections with *I* > 2σ(*I*)
*R*_int_ = 0.049

#### Refinement

*R*[*F* ^2^ > 2σ(*F* ^2^)] = 0.120
*wR*(*F* ^2^) = 0.347
*S* = 1.15
2877 reflections
215 parameters
H atoms treated by a mixture of independent and constrained refinement
Δρ_max_ = 1.44 e Å^−3^
Δρ_min_ = −0.46 e Å^−3^

### 

Data collection: *APEX2* (Bruker, 2007 ▶); cell refinement: *SAINT* (Bruker, 2007 ▶); data reduction: *SAINT*; program(s) used to solve structure: *SHELXS97* (Sheldrick, 2008 ▶); program(s) used to refine structure: *SHELXL97* (Sheldrick, 2008 ▶); molecular graphics: *ORTEP-3* (Farrugia, 1997 ▶); software used to prepare material for publication: *SHELXL97*.

## Supplementary Material

Crystal structure: contains datablock(s) global, I. DOI: 10.1107/S1600536812039529/zj2094sup1.cif

Structure factors: contains datablock(s) I. DOI: 10.1107/S1600536812039529/zj2094Isup2.hkl

Supplementary material file. DOI: 10.1107/S1600536812039529/zj2094Isup3.cml

Additional supplementary materials:  crystallographic information; 3D view; checkCIF report

## Figures and Tables

**Table 1:** Hydrogen-bond geometry (Å, °)

*D*—H⋯*A*	*D*—H	H⋯*A*	*D*⋯*A*	*D*—H⋯*A*
C16—H16*A*⋯O2^i^	0.96	2.59	3.468 (13)	153

Symmetry code: (i) 
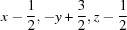
.

**Fig. 1.**
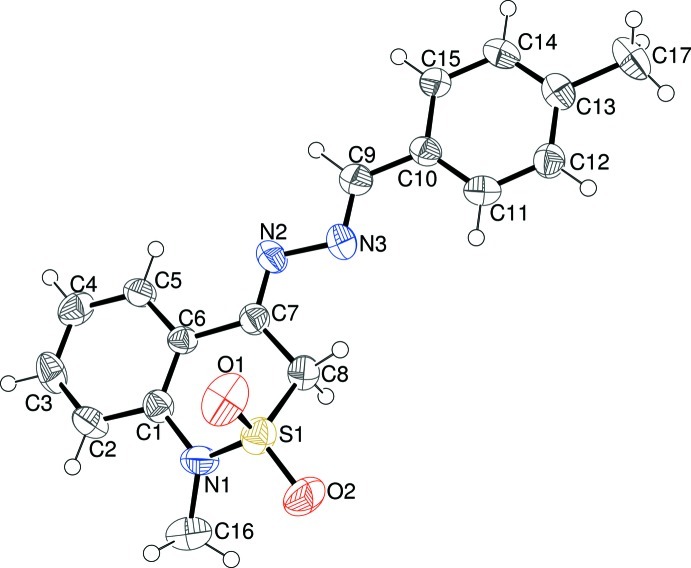
The molecular structure of (I), showing displacement ellipsoids at the 50% probability level.

**Fig. 2.**
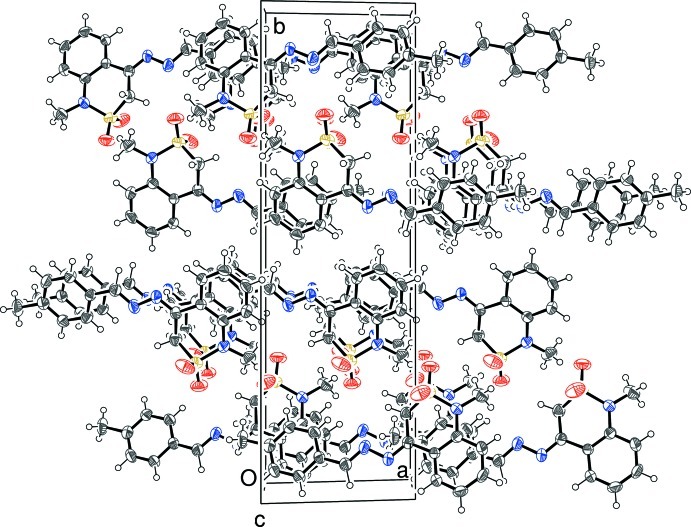
Detail of the packing of (I) showing part of a [101] chain of molecules linked by C—H···O hydrogen bonds (double dashed lines) and consolidated by aromatic π–π stacking between the centroids of the benzene and toluene rings (open pink lines). Symmetry codes: (i) -1/2 + x, 3/2 - y, -1/2 + z; (ii) -1 + x, y, -1 + z.

## supplementary crystallographic information

### Comment

As part of our ongoing studies of benzothiazine derivatives with potential biological activity (Shafiq, Zia-ur-Rehman *et al.*, 2011), we now describe the crystal structure of the title compound, (I), (Fig. 1).

The dihedral angle between the aromatic rings (C1–C6 and C10–C15) in (I) is 6.3 (5)° and the C7═N2—N3═C9 torsion angle is 179.8 (8)°. Similar values have been seen in related structures (Shafiq, Khan *et al.*, 2011; Shafiq *et al.*, 2012). The conformation of the thiazine ring in (I) is an envelope, with the S atom displaced by 0.823 (9) Å from the mean plane of the other five atoms (r.m.s. deviation = 0.012 Å). Again, this is similar to the situation in related structures (Shafiq, Khan *et al.*, 2011; Shafiq *et al.*, 2012).

In the crystal of (I) (Fig. 2), weak C—H···O interactions (Steiner, 2006) (Table 1) link the molecules to generate C(5) chains propagating in [101]. The chains are consolidated by weak aromatic π–π stacking between the benzene and toluene rings [centroid-centroid separation = 3.826 (5) Å, inter-planar angle = 6.3 (4)°].

### Experimental

For the synthesis, see: Shafiq, Zia-ur-Rehman *et al.* (2011). Yellow blades were recrystallized from ethyl acetate. The crystal quality was poor, which may correlate with the rather high residuals.

### Refinement

The H atoms were placed in calculated positions (C—H = 0.93–0.97 Å) and refined as riding. The methyl group was allowed to rotate, but not to tip, to best fit the electron density. The constraint *U*_iso_(H) = 1.2*U*_eq_(C) or 1.5*U*_eq_(methyl C) was applied. The highest difference peak is 1.27 Å from atom O2.

### Crystal data

**Table:**

C_17_H_17_N_3_O_2_S	*F*(000) = 688
*M**_r_* = 327.40	*D*_x_ = 1.370 Mg m^−^^3^
Monoclinic, *P*2_1_/*n*	Mo *K*α radiation, λ = 0.71073 Å
Hall symbol: -P 2yn	Cell parameters from 3678 reflections
*a* = 7.899 (1) Å	θ = 2.8–24.9°
*b* = 25.061 (3) Å	µ = 0.22 mm^−^^1^
*c* = 8.1743 (11) Å	*T* = 296 K
β = 101.114 (9)°	Blade, yellow
*V* = 1587.8 (3) Å^3^	0.45 × 0.21 × 0.09 mm
*Z* = 4	

### Data collection

**Table:**

Bruker APEXII CCD diffractometer	1897 reflections with *I* > 2σ(*I*)
Radiation source: fine-focus sealed tube	*R*_int_ = 0.049
Graphite monochromator	θ_max_ = 25.3°, θ_min_ = 2.7°
ω scans	*h* = −9→9
13483 measured reflections	*k* = −30→30
2877 independent reflections	*l* = −9→9

### Refinement

**Table:**

Refinement on *F*^2^	Secondary atom site location: difference Fourier map
Least-squares matrix: full	Hydrogen site location: inferred from neighbouring sites
*R*[*F*^2^ > 2σ(*F*^2^)] = 0.120	H atoms treated by a mixture of independent and constrained refinement
*wR*(*F*^2^) = 0.347	*w* = 1/[σ^2^(*F*_o_^2^) + (0.0894*P*)^2^ + 15.6763*P*] where *P* = (*F*_o_^2^ + 2*F*_c_^2^)/3
*S* = 1.15	(Δ/σ)_max_ < 0.001
2877 reflections	Δρ_max_ = 1.44 e Å^−^^3^
215 parameters	Δρ_min_ = −0.46 e Å^−^^3^
0 restraints	Extinction correction: *SHELXL97* (Sheldrick, 2008), Fc^*^=kFc[1+0.001xFc^2^λ^3^/sin(2θ)]^-1/4^
Primary atom site location: structure-invariant direct methods	Extinction coefficient: 0.011 (3)

### Special details

**Table:**

Geometry. All e.s.d.'s (except the e.s.d. in the dihedral angle between two l.s.planes) are estimated using the full covariance matrix. The cell e.s.d.'s are taken into account individually in the estimation of e.s.d.'s in distances, angles and torsion angles; correlations between e.s.d.'s in cell parameters are only used when they are defined by crystal symmetry. An approximate (isotropic) treatment of cell e.s.d.'s is used for estimating e.s.d.'s involving l.s. planes.
Refinement. Refinement of *F*^2^ against ALL reflections. The weighted *R*-factor *wR* and goodness of fit *S* are based on *F*^2^, conventional *R*-factors *R* are based on *F*, with *F* set to zero for negative *F*^2^. The threshold expression of *F*^2^ > σ(*F*^2^) is used only for calculating *R*-factors(gt) *etc*. and is not relevant to the choice of reflections for refinement. *R*-factors based on *F*^2^ are statistically about twice as large as those based on *F*, and *R*-factors based on ALL data will be even larger.

### Fractional atomic coordinates and isotropic or equivalent isotropic displacement parameters (Å^2^)

**Table:**

	*x*	*y*	*z*	*U*_iso_*/*U*_eq_	
C1	0.2464 (11)	0.6374 (3)	0.1249 (10)	0.0393 (19)	
C2	0.0924 (11)	0.6173 (4)	0.0246 (12)	0.048 (2)	
H2	−0.0062	0.6384	0.0023	0.058*	
C3	0.0902 (12)	0.5668 (4)	−0.0386 (13)	0.052 (2)	
H3	−0.0107	0.5539	−0.1047	0.063*	
C4	0.2323 (12)	0.5349 (4)	−0.0072 (12)	0.050 (2)	
H4	0.2283	0.5005	−0.0500	0.060*	
C5	0.3802 (11)	0.5541 (3)	0.0875 (11)	0.042 (2)	
H5	0.4779	0.5326	0.1070	0.050*	
C6	0.3899 (10)	0.6050 (3)	0.1561 (10)	0.0329 (17)	
C7	0.5555 (10)	0.6233 (3)	0.2611 (10)	0.0378 (19)	
C8	0.5629 (11)	0.6777 (3)	0.3398 (11)	0.046 (2)	
H8A	0.6805	0.6908	0.3583	0.055*	
H8B	0.5283	0.6751	0.4471	0.055*	
C9	0.9564 (11)	0.5776 (4)	0.4027 (12)	0.045 (2)	
H9	0.928 (13)	0.538 (4)	0.348 (12)	0.07 (3)*	
C10	1.1248 (10)	0.5891 (3)	0.5059 (10)	0.0362 (19)	
C11	1.1580 (11)	0.6354 (4)	0.5998 (11)	0.045 (2)	
H11	1.0710	0.6606	0.5980	0.054*	
C12	1.3211 (11)	0.6445 (3)	0.6965 (11)	0.044 (2)	
H12	1.3421	0.6755	0.7598	0.053*	
C13	1.4514 (11)	0.6077 (4)	0.6988 (11)	0.043 (2)	
C14	1.4177 (11)	0.5616 (4)	0.6071 (12)	0.051 (2)	
H14	1.5050	0.5365	0.6101	0.062*	
C15	1.2550 (10)	0.5515 (4)	0.5095 (10)	0.041 (2)	
H15	1.2343	0.5202	0.4480	0.049*	
C16	0.0902 (13)	0.7157 (4)	0.2205 (13)	0.059 (3)	
H16A	0.0280	0.7308	0.1185	0.089*	
H16B	0.1207	0.7435	0.3017	0.089*	
H16C	0.0189	0.6899	0.2617	0.089*	
C17	1.6295 (12)	0.6184 (5)	0.8015 (14)	0.064 (3)	
H17A	1.7116	0.6205	0.7292	0.096*	
H17B	1.6610	0.5900	0.8802	0.096*	
H17C	1.6284	0.6516	0.8602	0.096*	
S1	0.4268 (3)	0.72269 (9)	0.2121 (3)	0.0472 (7)	
N1	0.2450 (9)	0.6901 (3)	0.1900 (11)	0.052 (2)	
N2	0.6832 (9)	0.5911 (3)	0.2813 (9)	0.0448 (18)	
N3	0.8324 (9)	0.6113 (3)	0.3859 (10)	0.0477 (19)	
O1	0.4836 (11)	0.7265 (3)	0.0588 (9)	0.069 (2)	
O2	0.4100 (10)	0.7710 (2)	0.3007 (9)	0.064 (2)	

### Atomic displacement parameters (Å^2^)

**Table:**

	*U*^11^	*U*^22^	*U*^33^	*U*^12^	*U*^13^	*U*^23^
C1	0.038 (5)	0.036 (4)	0.042 (5)	−0.003 (4)	0.004 (4)	0.001 (4)
C2	0.033 (5)	0.049 (5)	0.061 (6)	0.006 (4)	0.004 (4)	0.006 (4)
C3	0.038 (5)	0.050 (6)	0.062 (6)	−0.016 (4)	−0.009 (4)	0.003 (5)
C4	0.057 (6)	0.033 (5)	0.061 (6)	−0.008 (4)	0.016 (5)	−0.011 (4)
C5	0.032 (4)	0.039 (5)	0.054 (5)	0.004 (4)	0.006 (4)	−0.004 (4)
C6	0.031 (4)	0.032 (4)	0.037 (4)	0.000 (3)	0.009 (3)	0.000 (3)
C7	0.036 (4)	0.042 (5)	0.036 (5)	−0.001 (4)	0.007 (4)	−0.001 (4)
C8	0.042 (5)	0.051 (5)	0.043 (5)	−0.001 (4)	0.006 (4)	−0.006 (4)
C9	0.039 (5)	0.047 (5)	0.048 (5)	0.001 (4)	0.010 (4)	−0.004 (4)
C10	0.034 (4)	0.040 (4)	0.035 (4)	0.004 (3)	0.008 (4)	0.006 (3)
C11	0.042 (5)	0.045 (5)	0.051 (5)	0.007 (4)	0.015 (4)	0.000 (4)
C12	0.043 (5)	0.041 (5)	0.046 (5)	−0.003 (4)	0.004 (4)	−0.004 (4)
C13	0.038 (5)	0.050 (5)	0.040 (5)	−0.003 (4)	0.005 (4)	0.011 (4)
C14	0.036 (5)	0.055 (6)	0.062 (6)	0.013 (4)	0.006 (4)	0.000 (5)
C15	0.038 (5)	0.045 (5)	0.040 (5)	0.006 (4)	0.006 (4)	−0.004 (4)
C16	0.067 (7)	0.051 (6)	0.066 (7)	0.013 (5)	0.028 (5)	0.005 (5)
C17	0.034 (5)	0.086 (8)	0.065 (7)	−0.001 (5)	−0.004 (5)	0.013 (6)
S1	0.0551 (15)	0.0354 (12)	0.0503 (14)	−0.0034 (10)	0.0081 (11)	−0.0015 (10)
N1	0.038 (4)	0.040 (4)	0.078 (6)	0.005 (3)	0.010 (4)	−0.012 (4)
N2	0.030 (4)	0.048 (4)	0.053 (5)	−0.001 (3)	0.001 (3)	−0.004 (3)
N3	0.035 (4)	0.054 (5)	0.052 (5)	−0.006 (3)	0.002 (3)	−0.007 (4)
O1	0.099 (6)	0.059 (4)	0.057 (4)	−0.025 (4)	0.033 (4)	0.004 (3)
O2	0.081 (5)	0.037 (3)	0.073 (5)	0.004 (3)	0.010 (4)	−0.013 (3)

### Geometric parameters (Å, º)

**Table:**

C1—C6	1.378 (11)	C10—C15	1.391 (11)
C1—C2	1.421 (12)	C11—C12	1.394 (12)
C1—N1	1.424 (11)	C11—H11	0.9300
C2—C3	1.366 (13)	C12—C13	1.378 (12)
C2—H2	0.9300	C12—H12	0.9300
C3—C4	1.362 (13)	C13—C14	1.377 (13)
C3—H3	0.9300	C13—C17	1.517 (12)
C4—C5	1.359 (12)	C14—C15	1.398 (12)
C4—H4	0.9300	C14—H14	0.9300
C5—C6	1.388 (11)	C15—H15	0.9300
C5—H5	0.9300	C16—N1	1.445 (11)
C6—C7	1.493 (11)	C16—H16A	0.9600
C7—N2	1.278 (10)	C16—H16B	0.9600
C7—C8	1.502 (12)	C16—H16C	0.9600
C8—S1	1.757 (9)	C17—H17A	0.9600
C8—H8A	0.9700	C17—H17B	0.9600
C8—H8B	0.9700	C17—H17C	0.9600
C9—N3	1.280 (11)	S1—O1	1.414 (7)
C9—C10	1.459 (12)	S1—O2	1.430 (6)
C9—H9	1.10 (10)	S1—N1	1.632 (8)
C10—C11	1.390 (12)	N2—N3	1.410 (9)
			
C6—C1—C2	118.7 (7)	C13—C12—C11	120.3 (8)
C6—C1—N1	122.8 (7)	C13—C12—H12	119.8
C2—C1—N1	118.4 (7)	C11—C12—H12	119.8
C3—C2—C1	119.5 (8)	C14—C13—C12	119.3 (8)
C3—C2—H2	120.2	C14—C13—C17	120.8 (9)
C1—C2—H2	120.2	C12—C13—C17	120.0 (9)
C4—C3—C2	121.5 (8)	C13—C14—C15	121.5 (8)
C4—C3—H3	119.2	C13—C14—H14	119.3
C2—C3—H3	119.2	C15—C14—H14	119.3
C5—C4—C3	119.1 (8)	C10—C15—C14	119.0 (8)
C5—C4—H4	120.4	C10—C15—H15	120.5
C3—C4—H4	120.4	C14—C15—H15	120.5
C4—C5—C6	122.0 (8)	N1—C16—H16A	109.5
C4—C5—H5	119.0	N1—C16—H16B	109.5
C6—C5—H5	119.0	H16A—C16—H16B	109.5
C1—C6—C5	119.2 (7)	N1—C16—H16C	109.5
C1—C6—C7	121.6 (7)	H16A—C16—H16C	109.5
C5—C6—C7	119.3 (7)	H16B—C16—H16C	109.5
N2—C7—C6	117.5 (7)	C13—C17—H17A	109.5
N2—C7—C8	123.6 (8)	C13—C17—H17B	109.5
C6—C7—C8	118.9 (7)	H17A—C17—H17B	109.5
C7—C8—S1	111.0 (6)	C13—C17—H17C	109.5
C7—C8—H8A	109.4	H17A—C17—H17C	109.5
S1—C8—H8A	109.4	H17B—C17—H17C	109.5
C7—C8—H8B	109.4	O1—S1—O2	117.9 (4)
S1—C8—H8B	109.4	O1—S1—N1	111.0 (5)
H8A—C8—H8B	108.0	O2—S1—N1	108.3 (4)
N3—C9—C10	121.7 (8)	O1—S1—C8	107.9 (5)
N3—C9—H9	118 (5)	O2—S1—C8	110.4 (4)
C10—C9—H9	120 (5)	N1—S1—C8	99.7 (4)
C11—C10—C15	119.6 (8)	C1—N1—C16	123.0 (8)
C11—C10—C9	122.5 (8)	C1—N1—S1	115.8 (6)
C15—C10—C9	117.9 (8)	C16—N1—S1	120.9 (6)
C10—C11—C12	120.3 (8)	C7—N2—N3	113.4 (7)
C10—C11—H11	119.8	C9—N3—N2	111.1 (7)
C12—C11—H11	119.8		
			
C6—C1—C2—C3	−0.3 (13)	C11—C12—C13—C17	178.7 (8)
N1—C1—C2—C3	−179.7 (9)	C12—C13—C14—C15	1.1 (14)
C1—C2—C3—C4	0.4 (15)	C17—C13—C14—C15	−178.9 (9)
C2—C3—C4—C5	−0.9 (15)	C11—C10—C15—C14	−0.5 (12)
C3—C4—C5—C6	1.3 (14)	C9—C10—C15—C14	179.8 (8)
C2—C1—C6—C5	0.7 (12)	C13—C14—C15—C10	−0.2 (14)
N1—C1—C6—C5	−179.9 (8)	C7—C8—S1—O1	−60.6 (7)
C2—C1—C6—C7	−179.6 (8)	C7—C8—S1—O2	169.2 (6)
N1—C1—C6—C7	−0.3 (12)	C7—C8—S1—N1	55.4 (7)
C4—C5—C6—C1	−1.2 (13)	C6—C1—N1—C16	−153.1 (9)
C4—C5—C6—C7	179.1 (8)	C2—C1—N1—C16	26.3 (13)
C1—C6—C7—N2	−178.7 (8)	C6—C1—N1—S1	32.7 (11)
C5—C6—C7—N2	0.9 (11)	C2—C1—N1—S1	−147.9 (7)
C1—C6—C7—C8	2.5 (11)	O1—S1—N1—C1	57.5 (8)
C5—C6—C7—C8	−177.9 (8)	O2—S1—N1—C1	−171.6 (6)
N2—C7—C8—S1	148.3 (7)	C8—S1—N1—C1	−56.1 (7)
C6—C7—C8—S1	−33.0 (9)	O1—S1—N1—C16	−116.9 (8)
N3—C9—C10—C11	4.0 (13)	O2—S1—N1—C16	14.1 (9)
N3—C9—C10—C15	−176.4 (8)	C8—S1—N1—C16	129.5 (8)
C15—C10—C11—C12	0.3 (13)	C6—C7—N2—N3	−178.3 (7)
C9—C10—C11—C12	179.9 (8)	C8—C7—N2—N3	0.4 (12)
C10—C11—C12—C13	0.6 (13)	C10—C9—N3—N2	−179.7 (7)
C11—C12—C13—C14	−1.3 (13)	C7—N2—N3—C9	179.8 (8)

### Hydrogen-bond geometry (Å, º)

**Table:**

*D*—H···*A*	*D*—H	H···*A*	*D*···*A*	*D*—H···*A*
C16—H16*A*···O2^i^	0.96	2.59	3.468 (13)	153

Symmetry code: (i) *x*−1/2, −*y*+3/2, *z*−1/2.
